# MICA: A fast short-read aligner that takes full advantage of Many Integrated Core Architecture (MIC)

**DOI:** 10.1186/1471-2105-16-S7-S10

**Published:** 2015-04-23

**Authors:** Ruibang Luo, Jeanno Cheung, Edward Wu, Heng Wang, Sze-Hang Chan, Wai-Chun Law, Guangzhu He, Chang Yu, Chi-Man Liu, Dazong Zhou, Yingrui Li, Ruiqiang Li, Jun Wang, Xiaoqian Zhu, Shaoliang Peng, Tak-Wah Lam

**Affiliations:** 1 HKU-BGI Bioinformatics Algorithms and Core Technology Research Laboratory, The University of Hong Kong, Hong Kong; 2 School of Computer Science, National University of Defense Technology, 410073, China; 3 BGI-Shenzhen, Shenzhen, 518083, China; 4 Department of Computer Science, The University of Hong Kong, Hong Kong

## Abstract

**Background:**

Short-read aligners have recently gained a lot of speed by exploiting the massive parallelism of GPU. An uprising alterative to GPU is Intel MIC; supercomputers like Tianhe-2, currently top of TOP500, is built with 48,000 MIC boards to offer ~55 PFLOPS. The CPU-like architecture of MIC allows CPU-based software to be parallelized easily; however, the performance is often inferior to GPU counterparts as an MIC card contains only ~60 cores (while a GPU card typically has over a thousand cores).

**Results:**

To better utilize MIC-enabled computers for NGS data analysis, we developed a new short-read aligner MICA that is optimized in view of MIC's limitation and the extra parallelism inside each MIC core. By utilizing the 512-bit vector units in the MIC and implementing a new seeding strategy, experiments on aligning 150 bp paired-end reads show that MICA using one MIC card is 4.9 times faster than BWA-MEM (using 6 cores of a top-end CPU), and slightly faster than SOAP3-dp (using a GPU). Furthermore, MICA's simplicity allows very efficient scale-up when multiple MIC cards are used in a node (3 cards give a 14.1-fold speedup over BWA-MEM).

**Summary:**

MICA can be readily used by MIC-enabled supercomputers for production purpose. We have tested MICA on Tianhe-2 with 90 WGS samples (17.47 Tera-bases), which can be aligned in an hour using 400 nodes. MICA has impressive performance even though MIC is only in its initial stage of development.

**Availability and implementation:**

MICA's source code is freely available at http://sourceforge.net/projects/mica-aligner under GPL v3.

**Supplementary information:**

Supplementary information is available as "Additional File 1". Datasets are available at www.bio8.cs.hku.hk/dataset/mica.

## Introduction

With the rapid advance of sequencing technologies, there is continuously demand for faster and faster analysis. The recently announced Illumina HiSeq × Ten sequencing system promises to sequence 18,000 whole human genomes (30x) in one year (four such systems can sequence more genomes than in all of history), while cutting the cost to $1,000 each. To cater to such capacity, it is important to develop new analysis software to fully utilize available acceleration hardware in addition to the CPU. For example, SOAP3-dp[1] uses a graphics processing unit (GPU) and is a few times faster than mainstream CPU aligners and delivers higher sensitivity. Besides GPU, attention has also fallen on Intel's new product Many Integrated Core (MIC), a.k.a. Xeon Phi Co-processor. MIC was introduced in 2011. It is an acceleration device whose hardware and system-software architecture support its use for general purpose computing. It is well suited to software implementations where computations on many thousands of data items can be carried out independently in parallel. The latest product has 57-61 cores and 8 GB of memory in one board, providing ~1 TFlops. Two of the top ten supercomputers in TOP500 (which ranks the world's 500 most powerful supercomputers) are equipped with MIC (Tianhe-2 has 48,000 MIC boards, and Stampede has 6,400 boards).

Experience has shown, however, that it is not easy to build useful read alignment software using massive core architectures such as GPU and MIC. The apparent problem is that the most biologically relevant sequence-alignment algorithm[2,3] involves dynamic programming dependencies that are awkward to compute efficiently in parallel. The fastest GPU implementations of the algorithm to date relies on task parallelism[1], where each thread of execution computes an entire alignment independently of all other parallel threads. This, however, requires sequences to be aligned to have similar lengths to ensure balanced tasks distribution among cores. Another typical sequence alignment problem is that a short query sequence (100 to 250 bp) must be aligned with a comparatively long (3 Gbp or longer) reference sequence. Since a brute-force search for all candidate alignments in this setting would be computationally prohibitive, read aligners typically construct a list of candidate reference sequence locations within which potential alignments might be discovered. The size of a list depends on the complexity of the sequence to be aligned and this accounts for a significant proportion of the computational imbalance involved in read alignment using massive core architectures. Several high-throughput read aligners including BarraCUDA[4], CUSHAW[5] and SOAP3-dp[1] that utilize GPU acceleration have been developed in the past few years, but to this date, limited studies have been carried out on short read alignment using MIC. This paper introduces MICA, a new short-read aligner designed to fully utilize the computing power of MIC.

## Method

**Figure 1:**
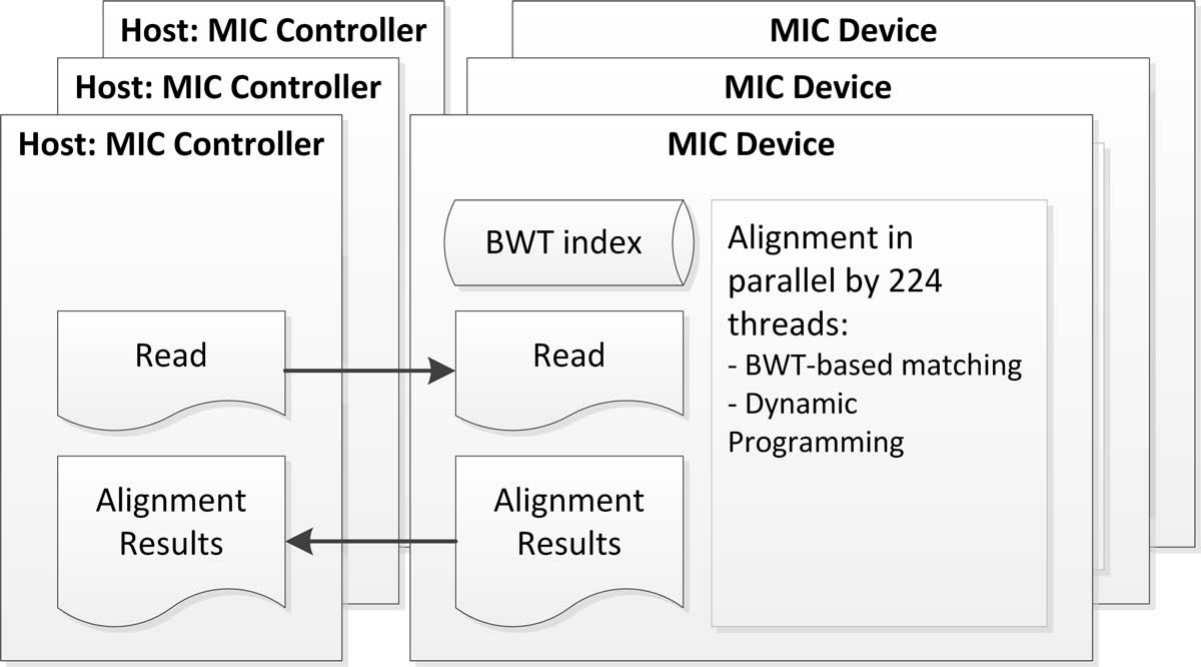
**MICA's architecture: interaction between the host and MIC cards**.

MICA accepts input reads in FASTA/FASTQ format and outputs alignment results in SAM/BAM format. In a typical setting, MICA runs on a server (host) equipped with 1 to 3 MIC cards. MICA runs each MIC using the offload mode (instead of native mode) to exploit the host's memory and provide better I/O performance. The latter is important when dealing with large volume of sequencing data. For each MIC card, MICA maintains a CPU thread in the host called an MIC-controller, which feeds the MIC with around million reads each time and spawns 224 MIC threads (running on 56 cores) to align the reads in parallel. At the end, the alignment results are copied to the MIC-controller for output (Figure 1). Note that reads with different complexities vary in aligning time. To achieve maximum throughput, MICA dynamically balances the load of the threads to avoid unnecessary idling.

Algorithmically, MICA maintains a BWT index in each MIC, and it adopts SOAP3-dp's approach to align reads shorter than 150 bp, and uses a new approach to handle longer reads. SOAP3-dp was designed for HiSeq 2000 paired-end reads of 100 bp; its efficiency stems from the fact that a GPU, with a 2 way-BWT index[6], can efficiently align at least one end of a paired-end read with 0-2 mismatches; the other end can be aligned using dynamic programming in the GPU. For longer reads (150 bp or more), SOAP3-dp deteriorates in sensitivity and speed because GPU is inefficient to align with more mismatches using the index. Thus, we need a new approach. In the following, we divide the discussion into two parts: the first part describes the techniques on how to utilize the resources of MIC to match the performance of SOAP3-dp on GPU, and the second part is about the new techniques for aligning reads.

Our experiment revealed that an MIC core was 4 to 6 times slower than a CPU core when running programs designed for CPU, and an MIC with 57 cores might be comparable to a 12-core CPU when used for brute-force parallelization of a CPU program. Noteworthy, each MIC core has thirty-two 512-bit registers; each allows sixteen 32-bit data to be operated in parallel. Such extra parallelism, if exploited properly, can boost the efficiency dramatically. This requires a lot of engineering work, though. Our first success is on the BWT index, MICA exploits 512-bit operations to speed up different arithmetic and memory transaction operations when querying the BWT (SOAP3-dp is based on 64-bit operations). Next, we turn to dynamic programming, which is for aligning reads allowing indels and soft clipping. Below we give the details of a new parallel algorithm for dynamic programming that can utilize the 512-bit registers.

### A. Highly parallel dynamic programming

**Figure 2:**
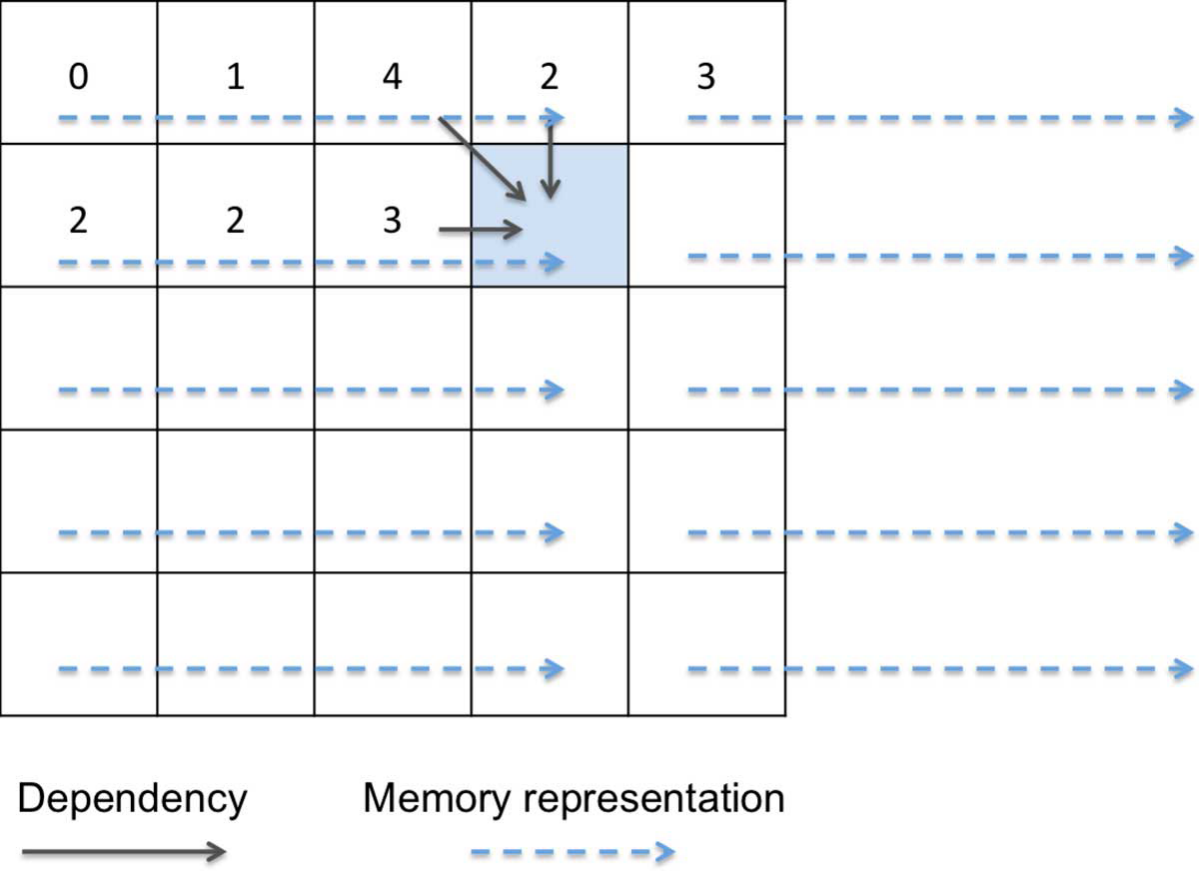
**Traditional approach to Dynamic Programming**.

To align a read of length *m *and a reference region of length *n *by DP, the traditional approach (Figure 2) needs to fill a 2-dimenional table (denoted *T*[*n*,*m*]), in which *T*[*i*, *j*] is determined by *T*[*i*-1,*j*-1], *T*[*i*-1,*j*] and *T*[*i*, *j*-1] (upper, upper-left and left dependencies). Such dependencies force us to compute consecutive entries on a row (or a column) one by one.

**Figure 3:**
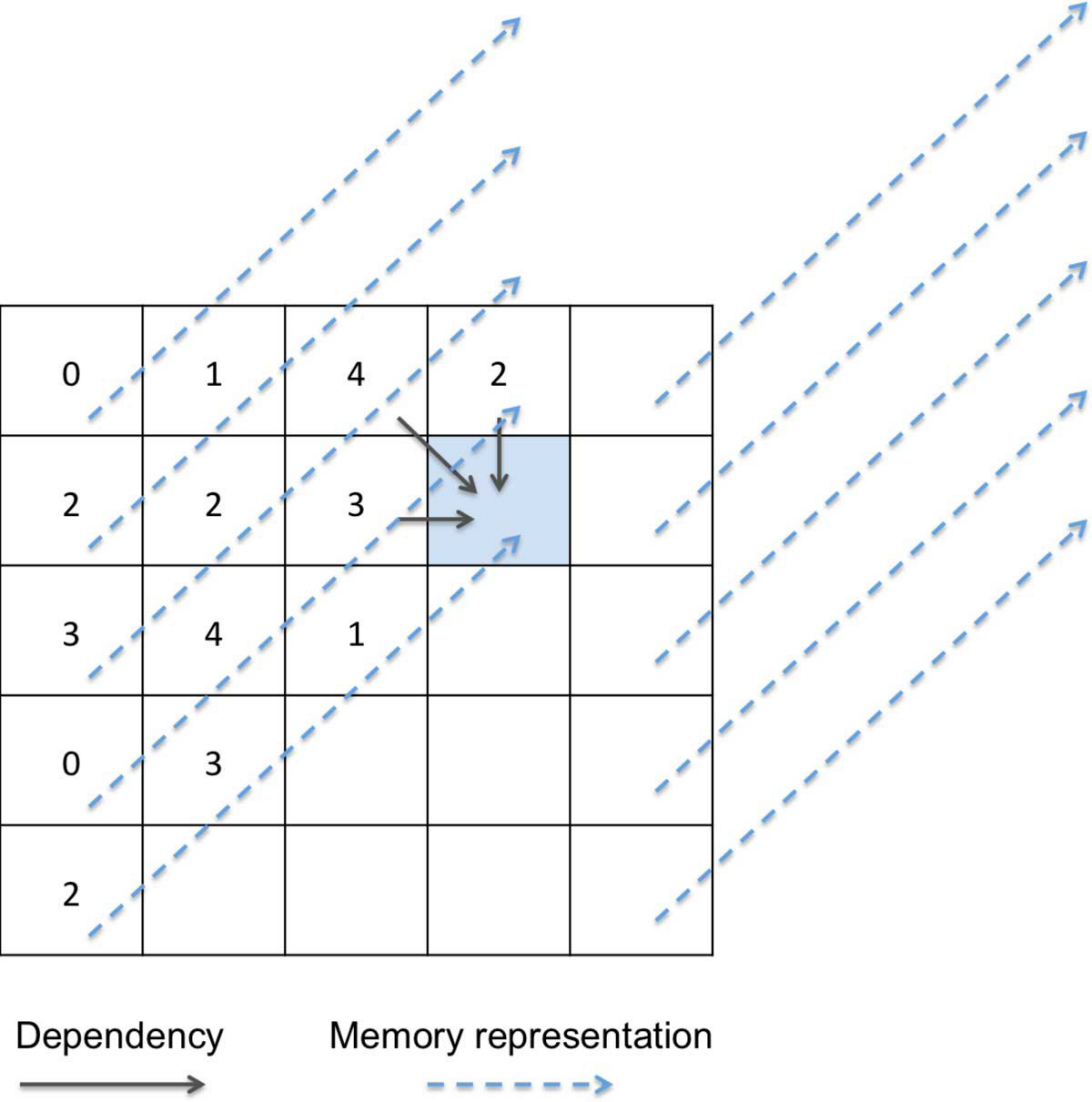
**MICA's approach to Dynamic Programming**.

To circumvent sequential dependencies, we represent this table in the diagonal order as a single array (Figure 3), i.e., *T*[*i*, *j*] is followed by *T*[*i*-1,*j*+1], *T*[*i*-2,*j*+2], and so on. This representation allows us to fill 16 entries of the table in one step. To fill *T*[*i*, *j*], *T*[*i*-1, *j*+1],..., *T*[*i*-15, *j*+15] in a single iteration, we need to load 16 entries from 3 memory locations beginning from *T*[*i*-1, *j*-*i*], *T*[*i*-1, *j*] and *T*[*i*, *j*-1]. Another concern is that the number of memory transactions in each core is an important factor to the overall efficiency. To minimize the number of memory transactions, for each entry, we pack all the necessary information (including the read and reference nucleotide) required for calculation into a 32-bit entry in the DP table and the memory transactions are reduced to loading only three 512-bit vectors of the DP table; The number "three" comes from the affine gap penalty model, which requires to compute three DP tables *M*, *I*, *D *to store the optimal score when aligning up to each position with the last character being a Match, Insert or Delete, respectively. To reduce memory transactions by increasing locality, the *M*, *I*, *D *values for each *i*, *j *are packed into *T*[*i*, *j*].

### B. New seeding strategies

For longer reads, we can only afford to use the BWT to align short fragments of a read (i.e., the seeds) and then count on dynamic programming to verify the candidate positions of the seed. Ideally we want fewer seeds (to save time on seed alignment) and the seeds should return correct candidate positions without too many incorrect ones (to save time on dynamic programming). MICA attempts to improve SOAP3-dp and other aligners by using as few seeds as possible, while maintains a balance between efficiency (fast & do not give too many candidates) and sensitivity (capture correct candidate positions for more reads). MICA uses a combination of the following strategies.

#### a) Non-branching mismatches

MICA allows more mismatches during seed alignment but without sacrificing efficiency. SOAP3-dp allows only one mismatch as the time for seed alignment increases exponentially with the number of mismatches (due to the branching of the search tree). We observe that very often a mismatch is unambiguous and there is exactly one way to correct it according to the reference genome. MICA allows 1-2 "non-branching" mismatches in addition to one "branching" mismatch for seed alignment.

**Figure 4:**
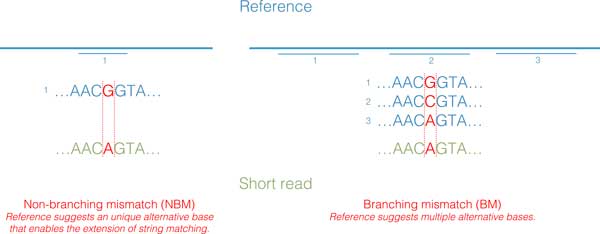
**Illustration of branching mismatches (BM) and non-branching mismatches (NBM)**.

In details, when performing alignment, we take a single character in the read and extend it character by character and check if the pattern exists in the reference genome. We call it a mismatch when we try to extend it such that the character in the read does not match the corresponding character in the reference genome. We can classify the mismatches into two types, branching mismatches (BM) and non-branching mismatches (NBM) (Figure 4). (a) When a NBM occurs, the current character in the read does not allow an extension, but there is another unique character that allows an extension. (b) When a BM occurs, there are at least 2 characters allowing an extension, one of them can be the character in the read, and we choose to extend it with a mismatch character.

For computing purposes, a BM increases the number of possible extensions much more than a NBM, and is more time-consuming to handle. Yet, we observed that very often a mismatch is unambiguous and there is exactly one way to correct it according to the reference genome (i.e. it is a NBM). Therefore, we allow MICA to find alignments with up to 2 non-branching mismatches, in addition to the 1 branching mismatch for seed alignment. This approach discovers more seeds than the "1 mismatch only" approach.

#### b) Read-sensitive seed length

MICA dynamically adjusts the seed length depending on the content of the read. If a region of the read appears to be non-repetitive, it uses a shorter seed to avoid missing critical candidate positions; for a region that appears to be repetitive, we choose a long seed to reduce the number of candidates.

More specifically, MICA follows SOAP3-dp's design for rapidly aligning those reads with < 3 mismatches and without an Indel, leaving those reads failed to be aligned (with ≥ 3 mismatches or with ≥ 1 Indel) to the Dynamic Programming (DP) module. This rapid mode works efficiently with 100 bp paired-end reads, where the slower DP module handles only about 10% of the reads. However, new sequencers such as Illumina HiSeq × Ten, produces paired-end reads 150 bp in length. The probability of a 150 bp read to span ≥ 3 mismatches or an Indel is higher than that of 100 bp, empirically forcing the DP module to handle 30-40% of the reads, which slows down the performance of MICA significantly. To enable MICA to work with 150 bp or longer reads efficiently, we have redesigned the workflow by disabling the rapid mode and using multiple rounds of seed discovery in the DP module to catch up to the speed. The longer seed lengths required in the former rounds limit the number of candidate alignment regions, where the shorter seed lengths in the latter rounds ensure the sensitivity, but requiring much more computation due to the large number of candidate alignment regions to be verified. Most reads with fewer mismatches and Indels thus could be aligned in the former rounds, leaving complicated reads for the latter rounds, which are more computationally demanding.

Table 1 gives the implementation details of each round as in the latest version of MICA. The details of SOAP3-dp are also provided for comparison.

**Table 1:** MICA and SOAP3-dp seeding details.

MICA:			
**Round**	**Seed Length**	**Seed Overlap**	**Seed # Limit**

Round 0	140	0	1,000

Round 1	80	0	1,000

Round 2	46	0	100

SOAP3-dp DP module:			

Round 1	28	20	100

Round 2	32	28	1,000

"Seed Overlap" means the maximum overlap between two seeds when extracting seeds from a read.

#### c) Breaking seeds at variant positions

MICA also tries to exclude read positions that are suspicious to be errors or variants (especially Indels) as part of a seed.

The above seeding strategies are used in multiple rounds with different parameters to attain better speed without sacrificing sensitivity. The superior sensitivity of the seeds allows MICA to pair up the candidate positions from both ends of almost all reads before the DP. This saves a lot of time on verifying incorrect positions.

## Results

### A. Software settings and machine specifications

*a) MICA: *Version r178. Compiled with Intel C/C++ Compiler version 13.1; one to three 57-core MIC cards (8G, ECC enabled), each coupled with a CPU core; and one CPU core for output (in SAM format).

*b) SOAP3-dp: *Version r176. Compiled with GCC 4.7.2 and CUDA SDK 5.5; one nVidia GeForce GTX680 (4G); 4 CPU cores; serial BAM output.

*c) BWA-MEM: *Version 0.7.5a. Compiled with GCC 4.7.2; 6 CPU cores. SAM output.

*d) In general: *Besides the acceleration devices (MIC/GPU), all other hardware is the same for all experiments. Specifically, Intel i7-3730k, 6-core @3.2 GHz, 64G memory

### B. Real data comparison with other aligners

We used 77-fold depth of 150 bp Illumina paired-end reads of the YH samples [7] (PE150) to benchmark MICA and other state-of-the-art aligners including GPU-based SOAP3-dp [1] and CPU-based BWA-MEM [8]. The results are shown in Table 2. The figures were calculated as the average of three repeated runs, and a tailor-made setting was used for each aligner to ensure the best performance. For the PE150 dataset, MICA using one card is ~4.9 times faster than BWA-MEM and slightly faster than SOAP3-dp. The speed of MICA can be scaled up almost linearly with additional cards. When using three cards, MICA is ~14.1 times faster than BWA-MEM. MICA's sensitivity is 3.1% and 0.47% higher than BWA-MEM and SOAP3-dp, respectively.

**Table 2:** Performance of MICA, SOAP3-dp and BWA-MEM on experimental data.

Dataset	Volume (Gbp)	# of Read Pairs (M)	Fold	MICA						SOAP3-dp		BWA-MEM	
				
				1 card	2 cards	2 cards scale-up	3 cards	3 cards scale-up	Properly paired	1 card	Properly paired	6 cores	Properly paired
PE150	232.15	773.83	77.38	20,919 s (5.81 hr)	10,618 s (2.95 hr)	1.97x	7,183 s (2.00 hr)	2.91x	95.48%	25,878 s (7.19 hr)	95.01%	101,466 s (28.19 hr)	92.32%

PE100	148.43	742.16	49.48	15,879 s (4.41 hr)	8,093 s (2.25 hr)	1.96x	5,453 s (1.51 hr)	2.91x	97.23%	17,982 s (5.00 hr)	97.08%	53,832 s (14.95 hr)	95.74%

We have also benchmarked 100 bp paired-end reads [7] (PE100, ~49-fold). Interestingly, MICA's acceleration on PE150 is slightly more significant than on PE100. For the latter, MICA is ~3.4 and ~9.9 times faster than BWA-MEM when using one and three MIC cards, respectively. When processing many shorter reads, MICA has a bottleneck in accessing the memory.

### A. Simulated data comparison with other aligners

We carried out simulated data test with short read simulator Mason[9] to assess the accuracy and sensitivity of MICA. We tested with 2 sets (100 bp and 150 bp) of 6M Illumina-style paired-end (PE) reads with 500 bp insert size from GRCh37 major build.

Bowtie2[10], SeqAlto[11] and GEM[12] allow users to sacrifice accuracy and sensitivity for speed with switches. We applied "very-fast", "sensitive", and "very-sensitive" switches to Bowtie2, "fast (-f)" to SeqAlto, and "fast adaptive (--fast-mapping)", "fastest (--fast-mapping = 0)" to GEM. We used indices with full suffix array (SA) for SOAP3-dp. All parameters of MICA, SOAP3-dp and SOAP3 were set to default, and parameters for other aligners including BWAaln and CUSHAW2[13,14] were set to favor the two read types and length. 13 sets of programs and parameters were compared in total.

In both 100 bp and 150 bp datasets, MICA is comparable to SOAP3-dp. MICA has advantage over other tools with higher speed, higher sensitivity and lower FDR results (Table 3 for 100 bp PE, and 150 bp table in supplementary).

**Table 3:** Comparison on 13 sets of programs and parameters using 100 bp paired-end simulated reads.

6M 100 bp Paired-end reads, 1.2 Gbp bases. 500 bp insert size, 25 bp standard deviation.			MIC	GPU		CPU									
			
			MICA(1 MIC Card, 240 threads)	SOAP3-dp	SOAP3	Bowtie2 (Sensititve)	Bowtie2 (Very-Sensititve)	Bowtie2 (Very-fast)	BWA^1^	SeqAlto	SeqAlto (Fast alignement)	CUSHAW2	GEM^2^	GEM^2^ (Fast Mapping: adaptive)	GEM^2^ (Fast Mapping: 0)
Configuration	CPU (thread: core i7-3930k)		**1**	4	4	4	4	4	4	4	4	4	4	4	4
	
	GPU (device: GTX680)		**0**	1	1	0	0	0	0	0	0	0	0	0	0

Computational Resources	Total Elapsed	sec.	**178**	162	132	966	1974	672	1154	495	379	1303	416	446	298
		
		Fold	**0.76**	1.23	1.00	7.32	14.95	5.09	8.74	3.75	2.87	9.87	3.15	3.38	2.26
	
	Loading Index^3^	sec.	**83**	74	74	38	38	38	53+1+1	96	96	40	40+1	40+1	40+1
	
	Alignment^4^	sec.	**95**	88	58	928	1936	634	370+369+360	399	283	1263	199+176	238+167	90+167
		
		Fold	**0.95**	0.88	0.58	9.28	19.36	6.34	10.99	3.99	2.83	12.63	3.75	4.05	2.57
	
	Avg. Memory	GB	**25.2**	17.2	17.3	3.3	3.3	3.3	3.5	7	6.9	3.6	4.3	4.3	4.3
	
	Peak Memory	GB	**29.9**	18.1	19.2	3.5	3.5	3.5	4.8	7.2	7.2	3.6	4.3	4.3	4.3

Alignment Metrics	Aligned	#	**11,999,810 **	11,999,827	11,870,740	11,999,763	11,999,936	11,998,226	11,998,804	12,000,000	11,995,872	11,999,975	11,999,763	11,999,484	11,995,422
		
		Diff.	**-**	17	-129,070	-47	126	-1,584	-1,006	190	-3,938	165	-47	-326	-4,388
	
	Properly Paired	#	**11999668**	11,999,460	11,742,902	11,998,912	11,999,344	11,996,528	11,997,254	11,999,976	11,995,410	11,977,218	11,998,994	11,997,702	11,991,992
		
		Diff.	**-**	-208	-256766	-756	-324	-3140	-2414	308	-4258	-22450	-674	-1966	-7676
	
	Incorrectly Aligned	#	**48,184 **	40,561	138,655	143,012	141,373	147,764	85,297	95,672	99,243	99,243	56,514	61,642	61,887
		
		Diff.	**-**	-7,623	90,471	94,828	93,189	99,580	37,113	47,488	51,059	51,059	8,330	13,458	13,703
	
	Sensitivity^5^	%	**99.60%**	99.66%	97.77%	98.81%	98.82%	98.75%	99.28%	99.20%	99.14%	99.17%	99.53%	99.48%	99.45%
		
		Diff.	**-**	0.06%	-1.83%	-0.79%	-0.78%	-0.85%	-0.32%	-0.40%	-0.46%	-0.43%	-0.07%	-0.12%	-0.15%
	
	FDR^6^	%	**0.40%**	0.34%	1.17%	1.19%	1.18%	1.23%	0.71%	0.80%	0.83%	0.83%	0.47%	0.51%	0.52%
		
		Diff.	**-**	-0.06%	0.77%	0.79%	0.78%	0.83%	0.31%	0.40%	0.43%	0.43%	0.07%	0.11%	0.12%

^1 ^The time consumption of BWA is calculated as "align left reads"+"align right reads"+"sampe". The index loading times of "align right reads" and "sampe" modules are 1 second due to the reason that, index files were cached during "align left reads". However, datasets larger than the host memory will flush the cache during alignment.

^2 ^The alignment time consumption of GEM is calculated as "alignment"+"convert to SAM format". The conversion module was run with 4 threads in consistent with the alignment module.

^3 ^SOAP3-dp, SOAP3, SeqAlto and GEM aligners explicitly provide index loading time consumption. The index loading time for Bowtie2, CUSHAW2 and BWA are calculated by the total size of index, divided by 100 MB/s, which is the average network file system speed of the testing environment. The index loading time maybe underestimated while the time processing the index was not calculated.

^4 ^The alignment times were explicitly provided by the aligners (include results processing and input/output time) or calculated by total elapsed time minus estimated index loading time.

^5 ^Sensitivity is calculated as "Correctly aligned reads"/"All simulated reads". The higher the better.

^6 ^FDR is calculated as "Incorrectly aligned reads"/"All aligned reads". The lower the better

For 100 bp reads, MICA takes 178 seconds to align 6M read pairs, which is 16 seconds slower than SOAP3-dp (due to the increasing time in loading the index), but 1.67 to 11.09 times faster than other tools. MICA's sensitivity is 99.60% which is 0.06% lower than SOAP3-dp but 0.07-1.83% higher than other tools. MICA's FDR is 0.40%, which is 0.07-0.79% lower than other tools except being 0.06% higher than SOAP3-dp.

### B. Production test on Tianhe-2 supercomputer

Tianhe-2 is currently the champion of the Top500 list of supercomputers. It has in total 16,000 computing nodes, each equipped with two 6-core Intel E5-2692 v2 @ 2.20 Hz CPU, 64 GB main memory and 3 MIC cards. Each MIC card has 57 cores and 8 GB on-board memory with ECC enabled.

To test Tianhe-2 with the workload of a population scale study, we used whole genome sequencing data of 45 CHB and 45 CHS samples (Supplementary Details) from the 1000 genomes project; a sample had 64.68-fold depth on average. In total, we had 932 lanes of 100 bp paired-end reads with size varying from 1.82- to 12.7-fold per lane.

The alignments of 932 lanes were carried out with 5 different settings. All alignment jobs were submitted simultaneously using one node per lane, occupying 4660 nodes in total at the very beginning. The results are shown in Table 4. 19 jobs failed to complete due to node or card failure, thus excluded from calculation. Using 3 cards per node and SAM output, the mean time consumption for 923 completed lanes is 1,303 seconds, with the largest lane finished within an hour. By stacking up the alignment jobs to minimize the idle time of computing nodes involved, we anticipate that all 923 jobs could be finished in an hour using less than 400 nodes.

**Table 4:** Results of experiments on Tianhe-2 using 5 different settings.

Setting	MIC card used	Output Format	Finished lane	Longest (sec)	Mean (sec)	Median (sec)
1	3	SAM	923	3,425	1,303	1,220

2	3	BAM	929	6,012	2,777	2,666

3	3	6-thread BAM	928	4,484	1,371	1,321

4	2	6-thread BAM	931	4,269	1,508	1,475

5	1	6-thread BAM	930	6,915	2,943	2,879

Using 3 cards and 6-thread BAM output, the mean time consumption is about the same but the maximum time consumption is ~18 minutes longer. Interestingly, alignment using 2 cards is almost twice as fast as using 1 card, but using 3 cards is only slightly faster than using 2 cards, since the computation was throttled by the slow compression algorithm in the BAM output module. Splitting the output into two or more files to enable non-blocking parallel compression will help in exploiting the full power of all three cards.

## Competing interests

The authors declare that they have no competing interests.

## Authors' contributions

S. C., J. C., E. W. and H. W. contributed equally to this work. Ruib. L. and T. L. managed the project and led the design. S. C., J. C., E. W. and H. W. implemented the software. W. L., D. Z., C. Y., C. L., Y. L., Ruiq. L., J. W., X. Z. and S. P. tested the software. S. C., J. C., E. W., Ruib. L. and T. L. wrote the paper.

## Declarations

The publication costs for this article were funded by Hong Kong GRF HKU-713512E and ITF GHP/011/12.

This article has been published as part of *BMC Bioinformatics *Volume 16 Supplement 7, 2015: Selected articles from The 11th Annual Biotechnology and Bioinformatics Symposium (BIOT-2014): Bioinformatics. The full contents of the supplement are available online at http://www.biomedcentral.com/bmcbioinformatics/supplements/16/S7.
